# Childhood pheochromocytoma crisis complicated with brain stem infarction: A case report

**DOI:** 10.1097/MD.0000000000032479

**Published:** 2022-12-23

**Authors:** Fujing Xie, Qingbing Zhao, Wenwen Pan, Anqi Zhang, Ke Li

**Affiliations:** a Department of Pediatrics, Liaocheng People’s Hospital, Liaocheng, Shandong Province, People’s Republic of China; b Department of Pediatrics, Dongchang Fu People’s Hospital, Liaocheng, Shandong Province; c Department of Central Laboratory, Liaocheng People’s Hospital, Liaocheng, Shandong Province, People’s Republic of China.

**Keywords:** autopsy, brain stem infarction, cerebral infarction, pheochromocytoma crisis

## Abstract

Pheochromocytoma crises are very rarely seen in children. In this report, we present a case of the death related to occult pheochromocytoma crisis combined cerebral infarction.

**Patient concerns:**

A 5-year-old boy has a 1-month history of polydipsia, polyuria, sweating, and weight loss of 2.5 kg. He was admitted to our hospital because of 1 week of anorexia, 2 days of vomiting, and 12 hours of convulsions and confusion. Magnetic resonance imaging of the brain and cervical spinal cord showed abnormal signals in the left parie-occipital lobe, medulla oblongata till C7 cervical vertebrae.

**Diagnoses:**

Based on patient’s complaints and clinical appearance, provisional diagnosis of pheochromocytoma crisis complicated brainstem infarction was considered.

**Interventions:**

Tracheal intubation, volume expansion, continuous infusion of dobutamine, and sedation reduce intracranial pressure. Chest compression was performed when the child suddenly developed sobbing respiration.

**Outcomes:**

The patient was dead. Congenital metabolic defects screening suggested mild ketonuria. Trio whole exon sequencing revealed a synonymous mutation of von Hippel–Lindau syndrome c.414 A > G in the decedent. Autopsy revealed pheochromocytoma, acute myocarditis, liquefaction necrosis of the medulla oblongata cerebral edema, and congestion.

**Lessons:**

Early clinical symptoms of pheochromocytoma in children are not typical. It may induce serious complications and develop into a pheochromocytoma crisis and cause death without proper treatment.

## 1. Introduction

Pheochromocytoma is a neuroendocrine tumor that arises in the chromaffin tissue of the adrenal medulla and sympathetic ganglia. It is rare in children with an estimated incidence of 2 per million. It induces abnormal secretion of catecholamines (epinephrine, norepinephrine, dopamine, etc) and leads to a constellation of clinical symptoms. As the clinical manifestations of pheochromocytoma could be vague and overlapping, diagnosis could be difficult and treatment is often delayed.

We report a case of the death related to occult pheochromocytoma crisis. The patient was diagnosed with pheochromocytoma crisis, brainstem infarction, and dysfunction of multiple organs including the heart, lung, and kidney, and finally died due to the lesion involving the medullary cardiovascular and respiratory control center. The autopsy confirmed the left adrenal pheochromocytoma, and genetic testing found a pathogenic mutation in von Hippel–Lindau syndrome (VHL) gene c.414 (exon2) A > G. To our knowledge, the combination of cerebral infarction was firstly reported in pediatric pheochromocytoma.

## 2. Case report

The 5-year-old boy, complaining of anorexia for 1 week, vomiting for 2 days, convulsions, and confusion for 12 hours, was admitted to the pediatric intensive care unit of Liaocheng People’s Hospital in January 2022. He had an average growth and development history, but suffered polydipsia, polyuria, sweating, nocturnal enuresis, and a weight loss of 2.5 kg in the past 1 month. The child had anorexia 1 week ago without abdominal pain and diarrhea, and occasional cough at night without fever. Two days ago, the child had projectile vomiting 4 times. He had fatigue but no complaints of headache, dizziness, or blurring of vision. Twelve hours ago, he had a sudden convulsion during sleep with turning up eyes, cyanotic lips, stiff legs and clenched fists, and did not respond to verbal stimuli. The symptoms resolved spontaneously after 30 seconds, but were followed by confusion, intermittent irritability, gibberish, shortness of breath, and gray and blotchy skin, and he was then admitted to our department. The child was the second birth of the family without obstetric abnormality, and had no documented history of head trauma or toxicant exposure, nor family history of hereditary diseases.

### 2.1. Admission vital signs

The patient had a temperature of 37°C, a heart rate (HR) of 156/min, respiration of 50/min, blood pressure (BP) reaching as low as undetectable, transcutaneous oxygen saturation of 88%, with a body weight of 23 kg. He had confusion, restlessness, gibberish, and shortness of breath. The patient showed cyanosis in the skin and lips; pupils were 3 mm, round, and responsive to light; the neck showed no resistance. He had difficulty breathing associated with supraclavicular retractions, suprasternal retractions, and sternal retractions. Lung sounds were coarse with rales. The HR was 156/min with a regular rhythm; the heart sound was muffled without murmur. The patient’s abdomen was soft, the liver was palpable 3 cm below the right costal margin, the spleen was not palpable, and the bowel sounds were normal. Distal parts of extremities were cool to elbows and knees. Capillary refill time was >5 seconds. The pathological reflex was negative.

### 2.2. Laboratory examinations

Further laboratory assessments are as follows: multiple blood gas measurements indicated respiratory alkalosis (pH 7.37–7.55, PaCO2 23.2–38.4 mm Hg), hyperlactatemia (maximum blood lactate concentration 5.7 mmol/L), and electrolyte imbalance. Complete blood count showed white blood cell 12.92 × 10^9^/L, percentage of neutrophils 79.50%. Blood chemistry panel exhibited reduced renal function with an increased blood urea nitrogen of 7.60 to 8.05 mmol/L (reference range 3.2–7.1 mmol/L) and electrolyte disorder: serum sodium 119.1 mmol/L, serum chlorine 87.0 mmol/L, and serum potassium 3.1 mmol/L. The minimum blood glucose was 2.5 mmol/L. Myocardial infarction biochemical markers were all significantly increased: troponin I 2.380 ng/mL (reference range 0–0.034 ng/mL), myoglobin 709.7 ng/mL (reference range 0–121 ng/mL), creatine kinase-MB 7.90 ng/mL (reference range 0–3.38 ng/mL), N-terminal-pro hormone BNP >35000 pg/mL (reference range 0–125 pg/mL). Cerebrospinal fluid analysis showed an elevated protein level of 0.85 g/L, nucleated cell count of 7 × 10^6^/L, and proportions of lymphocytes and activated monocytes were 19% and 68%, respectively.

Blood ammonia was normal; repeated urinalysis showed urine protein of 1 to 4 g/L and positive occult blood. Concentrations of D-dimer 1.49 mg/L (reference range 0–0.5 mg/L) and fibrinogen/fibrin degradation products 3.43 mg/L (reference range 0–2.01 mg/L) were elevated. Infection markers C-reaction protein and erythrocyte sedimentation rate 18 mm/Hr were regular, but procalcitonin was 1.78 ng/mL (reference range 0–0.05 ng/mL).

Pathogenic detection results are as follows. Toxoplasmosis, others(Syphilis, Hepatitis B), rubella, Cytomegalovirus (CMV), and herpes simplex antibody serological assays showed only IgM antibody for herpes simplex virus was high 1.47 (reference range ≤ 1.1); Epstein–Barr virus antibody profile results were all negative. Sputum bacteria culture detected the presence of *Streptococcus pneumonia*. The blood culture report was negative. Cerebrospinal fluid bacteria culture, acid-fast staining, India ink staining, and PCR detection for EBV, cytomegalovirus, Enterovirus 71 virus, coxsackievirus A16 and enterovirus universal ribonucleic acid detection were all negative. Respiratory nucleic acid detections of *Mycoplasma*, influenza A and B, and *Mycobacterium tuberculosis* were all negative.

### 2.3. Cardiac examinations

The electrocardiogram showed sinus tachycardia with ST-T-wave changes (152/min); the echocardiography showed ascending aorta dilation, left ventricular hypertrophy, segmental myocardial dyskinesia, and left ventricular systolic dysfunction (ejection fraction 33%).

### 2.4. Imaging examinations

**Figure 1.**
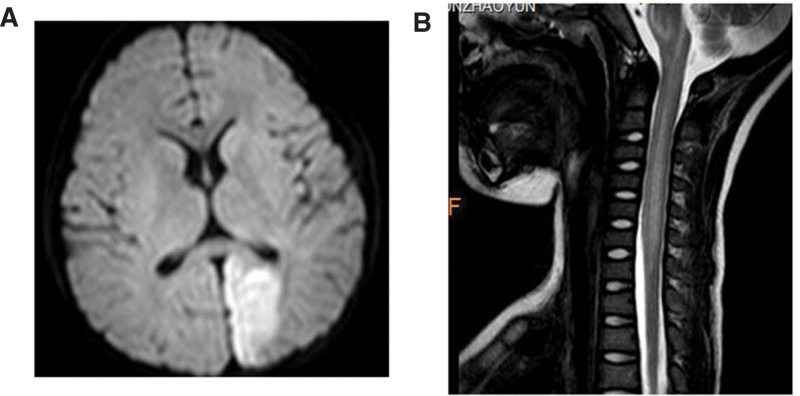
MRI imaging of the patient. (A) MRI of the brain showed patchy iso-intense T1 and iso-intense T2 signals in the left parietal occipital lobe. (B) MRI of the cervical spine showed longer T1 and T2 signals in the medulla oblongata to C7 cervical vertebrae with the presence of local edema. MRI = magnetic resonance imaging.

Chest X-ray showed bilateral exudative lesions of bilateral lungs. Magnetic resonance imaging of the brain and cervical spine showed patchy iso-intense T1 and iso-intense T2 signals in the left parietal occipital lobe (Fig. 1A), hyperintensity in fluid-attenuated inversion recovery sequences, restricted diffusion on diffusion-weighted imaging; longer T1 and T2 signals were observed in the medulla oblongata to C7 cervical vertebrae (Fig. 1B) with the presence of local edema.

### 2.5. Treatment

Upon admission, tracheal intubation was immediately implanted to facilitate ventilation with the setting as follows: fraction inhaled oxygen 45%, positive end-expiratory pressure 10 cm H2O, respiratory rate 25/min, PC >10 cm H2O, tidal volume 160 mL. Intravenous infusion included rapid infusion of 350 mL normal saline for volume expansion, continuous infusion of dobutamine (5.8 µ/kg/min) to improve cardiac contractility and output, midazolam (2.6 µ/kg/min) for sedation, mannitol (100 mL q.4.h.) to reduce elevated intracranial pressure, vitamin C 4g and fructose sodium diphosphate 3g to nourish myocardium, cefoperazone/sulbactam 1.5 g to prevent infection, and 3% sodium chloride (70 mL) to correct hyponatremia. In preparation for mechanical circulatory support, a femoral catheter was inserted.

The patient was in a coma with a fever (37.9–40°C), HR 135 to 150/min, and BP 94 to 131/56 to 72 mm Hg. At 10.5 hours after admission, the child suddenly developed sobbing respiration, HR decreased to 60/min, and transcutaneous oxygen saturation decreased to 60%. Chest compression was immediately performed, ventilator fraction inhaled oxygen was set to 100%, an intravenous bolus of 1:10000 epinephrine (0.01mg/kg) and norepinephrine (0.1–1 µg/kg/min) was given to raise BP, and sodium bicarbonate was administrated to treat acidosis. The child’s HR once rose to 160/min but quickly dropped to 0/min; normal sinus rhythm failed to spontaneous restoration; no spontaneous breathing; bilateral pupils dilated without light reflex; electrocardiogram was flatlined, and the patient was pronounced dead.

**Figure 2.**
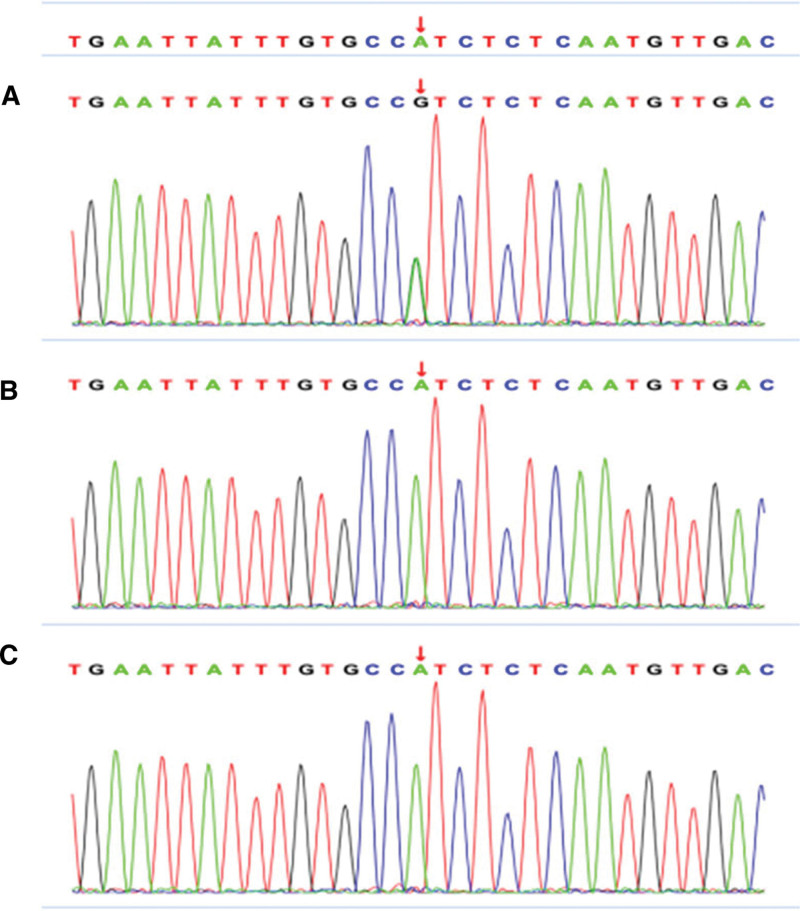
Trio whole exon sequencing (WES). (A) The patient had a synonymous mutation of VHL; (B–C) his mother and father did not bear this mutation. VHL = von Hippel–Lindau syndrome.

Informed consent from the patient’s family was obtained for further clinical investigation of the cause of death. Congenital metabolic defects screening (MilsLab Genetic Metabolic Mass-spec Center, China) on urine and blood samples suggested mild ketonuria. Trio whole exon sequencing (Chigene, Beijing, China) revealed a synonymous mutation of VHL c.414 A > G in the decedent, which is heterozygous de novo mutation; his parents did not bear this mutation and showed no symptoms, consistent with autosomal dominant inheritance (Fig. 2). According to the guidelines of the American College of Medical Genetics and Genomics, this variant is pathogenic and co-segregated with a total population frequency of 0, and was found associated with pheochromocytoma (OMIM:171300) and VHL (OMIM:193300).

### 2.6. Autopsy

Informed consent was obtained from the family for a “no restrictions” autopsy. Gross examination of organ systems revealed a left adrenal tumor which was diagnosed as pheochromocytoma based on morphology, acute myocarditis involving left and right ventricle posterior wall and ventricular septal, edema and congestion in bilateral lungs, and chronic hepatic congestion. Examination of the brain showed widespread multifocal areas of liquefaction necrosis of the medulla oblongata, cerebral edema, and congestion.

## 3. Discussion

Pheochromocytoma is a neuroendocrine tumor that arises in the chromaffin tissue of the adrenal medulla and sympathetic ganglia. It is rare in children with an estimated incidence of 2 per million.^[1]^ It induces abnormal secretion of catecholamines (epinephrine, norepinephrine, dopamine, etc) and leads to a constellation of clinical symptoms.

The main manifestations include episodic or persistent hypertension and its secondary manifestations caused by a large amount of catecholamines entering the circulation, accompanied by headache, palpitations, sweating, paleness, nausea, and vomiting, which may completely remit. Studies have shown that childhood pheochromocytoma often presents with persistent hypertension, and episodic hypertension is rare.^[2]^ Severe hypertension can lead to optic nerve head edema, hemorrhage and small artery spasm and other fundus lesions, resulting in blurred or decreased vision. Persistent hypertension can cause cardiac hypertrophy, especially in the left ventricular, subsequently leading to hypertensive heart disease and congestive heart failure. Pheochromocytoma induces endocrine and metabolic disorders, which increase the basal metabolic rate, cause weight loss, and affect glucose metabolism, resulting in hyperglycemia, glycosuria, and abnormal glucose tolerance curve. Gastrointestinal symptoms include an abdominal pain, constipation, intestinal obstruction, and even acute abdominalgia due to tumor hemorrhagic necrosis. Polydipsia and polyuria can be caused by profuse sweating, or catecholamines’ suppression of antidiuretic hormone. Life-threatening conditions, that is, pheochromocytoma crisis, are combined with shock or compromises vital organ function. The incidence of crisis in pheochromocytoma is 7 to 18%^[3–5,7]^ which usually occurs in adults and is combined with myocardial infarction, cardiogenic shock and stroke.^[6–9]^ Reports of pheochromocytoma crises in children are rare.

We report a case of the death of a boy related to an occult pheochromocytoma crisis and possibly provoked cerebral infarction by catecholamines, and to our knowledge the combination of cerebral infarction was firstly reported in pediatric pheochromocytoma. Despite no history of hypertension, headache or palpitations provided, the young decedent showed symptoms of vomiting and fatigue, and echocardiography showed left ventricular hypertrophy, which suggested a history of hypertension. During the past month, the boy showed hyperhidrosis, polydipsia, polyuria, nocturnal enuresis, and a weight loss of 2.5 kg, suggesting an increased basal metabolic rate and involvement of the endocrine and metabolic disorders. The autopsy showed a left adrenal pheochromocytoma and the definitive diagnosis of childhood pheochromocytoma was made.

The patient was in shock upon admission, which is a composite result of multiple possible pathogeneses. Catecholamines secreted by the tumor provoked severe hypertension and the vagus nerve was reflexively excited; catecholamines may also cause strong vasoconstriction and tissue hypoxia, followed by increased microvascular wall permeability and plasma transudation, resulting in the decreased effective arterial blood volume; hemorrhage, necrosis, or embolism might occur in the tumor and cause a sudden decrease or termination of catecholamine secretion and consequently lead to BP dropping; the tumor might secrete a variety of vasodilators, such as vasoactive intestinal peptide and adrenomedullin; which may even cause the cardiogenic shock of the patient.

The young patient was found with multiorgan dysfunction. His cardiac manifestations included catecholaminergic cardiomyopathy, heart failure, and shock. Based on central nervous system manifestations and autopsy results, infarction of the brain and brain stem was considered, which may result from the considerable catecholamine secretion-led cerebral vasoconstriction, ischemia, and necrosis. The patient’s breathing symptoms and chest X-ray images indicated the affected medulla oblongata respiratory center and central neurogenic respiratory failure. On admission, the patient showed elevated blood urea nitrogen and normal creatinine, which was initially considered shock-induced prerenal kidney injury; considering the multiple positive urine protein, the renal damage might cause by pheochromocytoma hypertension. The child developed hemodynamic disturbance and multiorgan damage upon visiting the clinic; the diagnosis of pheochromocytoma crisis accompanied by brainstem infarction was clear after autopsy, and eventually respiratory and heart failure caused the death of this patient.

As the clinical manifestations of pheochromocytoma could be vague and overlapping, diagnosis could be difficult and treatment is often delayed. However, in this case, early recognition of hypertension or noticed upon seeking medical attention for hyperhidrosis, polydipsia, and polyuria, and improved serum/urine vanilmandelic acid detection and abdominal computed tomography would help in early diagnosis of pheochromocytoma. Early treatment by α-adrenergic receptor blockers, β-blockers or calcium channel blockers, followed by surgical resection, there still was a chance for the young patient to survive. Studies reported that the incidence of pheochromocytoma in children with hypertension is 0.8 to 1.7%.^[10,11]^ Compared to adults, pediatric pheochromocytoma is more possibly associated with genetic defects. The genetic examination of this case reported a de novo mutation of VHL c.414 (exon2) A > G, which is pathogenic and associated with pheochromocytoma and VHL.

Early clinical symptoms of pheochromocytoma in children are not typical. It may induce serious complications and develop into a pheochromocytoma crisis and cause death without proper treatment. Early diagnosis and timely medication and surgical treatment are the keys to reducing complications and improving survival.

## Author contributions

**Conceptualization:** Fujing Xie, Qingbing Zhao, Wenwen Pan.

**Data curation:** Fujing Xie, Qingbing Zhao, Wenwen Pan, Anqi Zhang, Ke Li.

**Formal analysis:** Qingbing Zhao, Wenwen Pan.

**Investigation:** Fujing Xie, Wenwen Pan.

**Resources:** Fujing Xie.

**Supervision:** Anqi Zhang.

**Validation:** Anqi Zhang, Ke Li.

**Writing – original draft:** Fujing Xie, Anqi Zhang.

**Writing – review & editing:** Ke Li.

## References

[1] Arbay O Cif Tci FT Anyel C. Pheochromocytoma in children. J Pediatr Surg. 2001;36:447–52. 11226993 10.1053/jpsu.2001.21612

[2] Perel Y Schlumberger M Marguerite G. Pheochromocytoma and praganglioma in children: a report of 24 cases of the French society of pediatric oncology. Pediatr Hematol Oncol. 1997;14:413–22. 9267873 10.3109/08880019709028771

[3] Scholten A Cisco RM Vriens MR. Pheochromocytoma crisis is not a surgical emergency. J Clin Endocrinol Metab. 2013;98:581–91. 23284003 10.1210/jc.2012-3020

[4] Whitelaw BC Prague JK Mustafa OG. Phaeochro mocytoma crisis. Clin Endocrinol (Oxf). 2014;80:13–22. 24102156 10.1111/cen.12324

[5] Riester A Weismann D Quinkler M. Life-threatening events in patients with pheochromocytoma. Eur J Endocrinol. 2015;173:757–64. 26346138 10.1530/EJE-15-0483

[6] Liu Z-G Luan C-Y Zhao P-T. Electrocardiograph resembles acute ST segment elevation myocardial infarction in pheochromocytoma crisis: a case report. Zhonghua Xin Xue Guan Bing Za Zhi. 2012;40:437–8. 22883100

[7] Ando Y Ono Y Sano A. Clinical characteristics and outcomes of pheochromocytoma crisis: a literature review of 200 cases. J Endocrinol Invest. 2022;45:2313–28. 35857218 10.1007/s40618-022-01868-6

[8] Sanna GD Talanas G Fiore G. Pheochromocytoma presenting as an acute coronary syndrome complicated by acute heart failure: the challenge of a great mimic. J Saudi Heart Assoc. 2016;28:278–82. 27688679 10.1016/j.jsha.2016.02.002 PMC5034488

[9] Bouabdallaoui N Bouchard D Jolicoeur EM. Extracorporeal membrane oxygenation in pheochromocytoma-induced cardiogenic shock. Asian Cardiovasc Thorac Ann. 2018;26:314–6. 28823181 10.1177/0218492317727995

[10] Cohen JK Cisco RM Scholten A. Pheochromocytoma crisis resulting in acute heart failure and cardioembolic stroke in a 37-year-old man. Surgery. 2014;155:726–7. 23305592 10.1016/j.surg.2012.11.013

[11] Lenders JW Duh QY Eisenhofer G. Endocrine Society. Pheochromocytoma and paraganglioma: an endocrine society clinical practice guideline. J Clin Endocrinol Metab. 2014;99:1915–42. 24893135 10.1210/jc.2014-1498

